# Effective healing of endoscopic submucosal dissection-induced ulcers by a single week of proton pump inhibitor treatment: a retrospective study

**DOI:** 10.1186/s13104-015-1111-2

**Published:** 2015-04-15

**Authors:** Shinya Kajiura, Ayumu Hosokawa, Akira Ueda, Hiroshi Mihara, Takayuki Ando, Haruka Fujinami, Jun Nishikawa, Kohei Ogawa, Masami Minemura, Toshiro Sugiyama

**Affiliations:** Department of Gastroenterology and Hematology, Faculty of Medicine, University of Toyama, 2630 Sugitani, Toyama-shi, Toyama-ken 930-0194 Japan; Department of Medical Oncology, University of Toyama, 2630 Sugitani, Toyama-shi, Toyama-ken 930-0194 Japan

**Keywords:** Endoscopic submucosal dissection, ESD-induced ulcers, Proton pump inhibitor

## Abstract

**Background:**

Although artificial ulcers generally heal faster than *Helicobacter pylori*-related or nonsteroidal anti-inflammatory drug-related peptic ulcers, endoscopic submucosal dissection (ESD)-induced gastric ulcers are usually treated with a proton pump inhibitor (PPI) for 4–8 weeks. The effect of oral administration of a PPI for 1 week on ESD-induced gastric ulcers has not yet been evaluated. In the present study, we evaluated the efficacy of oral PPI for 1 week in patients with ESD-induced ulcers.

**Methods:**

We selected 45 patients who underwent ESD for gastric mucosal tumors between June 2005 and July 2006 at Toyama University Hospital, and who met our inclusion criteria. All patients received omeprazole intravenously for 2 days after ESD and then orally for 1 week to prevent bleeding. Twenty two patients received no further omeprazole therapy (1-week group) and the rest received omeprazole orally for 7 more weeks (8-week group). Follow-up endoscopy was performed at 1 day, 4 weeks, and 8 weeks after ESD. We compared the ulcer healing rates between both groups.

**Results:**

There were no significant differences between the groups in the ulcer-healing rate, because ulcers healed in 22 (96%) and 20 (91%) patients from the 8-week and 1-week groups, respectively.

**Conclusions:**

In our study, oral administration of omeprazole for 1 week was sufficient to achieve healing of ESD-induced artificial gastric ulcers. A larger prospective trial will be required to confirm these findings.

## Background

Endoscopic mucosal resection (EMR) is a promising treatment for early gastric cancer because it is less invasive than surgery. EMR has been widely used as a curative treatment for gastric mucosal tumors such as early gastric cancer or adenoma [1,2]. Endoscopic submucosal dissection (ESD) is a new EMR method that is now being used to cure early gastric cancer and adenomas in Japan [3]. Because *en bloc* resection of gastric tumors is crucial for an accurate histological diagnosis to confirm complete resection, ESD is superior to EMR for treating large lesions. Improvements in endoscopic equipment and techniques have allowed *en bloc* resection of whole lesions (regardless of lesion size or shape) to become possible with circumferential cutting of the surrounding mucosa using the ESD method [4-6]. This method allows the precise histopathological diagnosis of the resected specimen; however, an ulcer induced by ESD is usually larger than that one induced by standard EMR [5].

Artificial gastric ulcers heal faster than *Helicobacter pylori* (*H. pylori*)-related or nonsteroidal anti-inflammatory drug (NSAID)-related gastric ulcers. However, the management of ulcers caused by ESD has not been fully investigated and more clinical data are needed [7]. Acid-suppressing agents are invariably administered to prevent bleeding and induce rapid healing of these ulcers. Gastric ulcers induced by ESD are usually treated with a proton pump inhibitor (PPI) for 4–8 weeks [8-10]. However, there is no consensus on the optimal duration of the PPI treatment, and no study yet has tested whether the use of a PPI for 1 week is sufficient to heal ESD-induced gastric ulcers. Therefore, we compared the healing rates of ESD-induced ulcers in patients who received orally administered omeprazole for 1 or 8 weeks.

## Methods

### Study design and patients

Among patients who underwent ESD for gastric mucosal tumors between June 2005 and July 2006 in Toyama University Hospital, we selected patients who met our inclusion criteria and did not meet our exclusion criteria. Indications for ESD included gastric adenoma or early gastric cancer (well-differentiated or moderately differentiated) of any size without lymph node involvement or other metastasis at the time of diagnosis. Inclusion criteria included the administration of 20 mg of omeprazole orally for 1 or 8 weeks after intravenous administration of 20 mg of omeprazole for 2 days after ESD. Exclusion criteria were as follows: (1) a history of upper gastrointestinal surgery; (2) serious complications, including cardiac, hematological, renal, and/or hepatic disease; (3) the recent use of PPI, a histamine 2 receptor blocker or a mucosal protective agent; (4) concurrent gastric/duodenal ulcer (excluding healed ulcers); (5) current use of aspirin, NSAIDs or corticosteroids; or (6) absence of written informed consent from a patient.

In all patients, ESD was performed using a needle knife and a hook knife (Olympus Optical Co., Ltd., Tokyo, Japan) with the injection of glycerin and hyaluronic acid solutions. All patients were administered omeprazole intravenously for 2 days after ESD and then orally for 1 week to prevent bleeding after ESD. However, because there was no consensus on the optimal duration of the PPI treatment, our hospital doctors had agreed to provide the PPI treatment for a minimum of 1 week after ESD. When this minimum treatment was chosen, the PPI treatment duration was 1 week. The duration of the PPI treatment was decided by each physician. Different treatment durations were chosen and treatment was implemented accordingly. To investigate the effect of the duration of the PPI treatment, we selected patients who underwent treatment for 1 or 8 weeks for this study. Thus, half of the patients received no further omeprazole therapy and the rest received an additional 7 weeks of omeprazole therapy. Age, gender, initial diagnosis, site of the lesion, *H. pylori* status and smoking habits of all patients were recorded before the ESD procedure. After the procedure, data regarding abdominal pain and bleeding were recorded for all the patients. The presence of *H. pylori* infection was assessed by performing a histological examination and the rapid urease test using four endoscopic biopsy specimens (2 from the antrum and 2 from the body of the stomach), as well as testing for serum *H. pylori* immunoglobulin G antibodies. We determined that a patient as had *H. pylori* infection when either of these tests was positive. In addition, we retrospectively analyzed data in the medical records of these patients. This study was approved by the Ethics Committee, University of Toyama (Approval number: Rin-nin No. 22–48).

### Evaluation of ulcer healing and symptoms

The size of the ulcer caused by ESD was determined by measuring the resected specimen as this provided a more accurate measurement than assessment of the ulcer in the stomach with an endoscope. Ulcer area was calculated as the maximum diameter multiplied by the perpendicular diameter. Follow-up endoscopy was performed at 1 day, 4 weeks, and 8 weeks after ESD. Ulcers were also staged according to a 6-stage classification system proposed by Sakita and Fukutomi (Table 1) [11]. The size of the ulcer at 4 and 8 weeks after ESD was estimated from the maximum diameter alone rather than by multiplying the 2 diameters, because the ulcers had decreased in size and it was difficult to measure the smaller perpendicular diameter.

**Table 1:** **Staging system of gastric ulcer**

**Stage**	**Findings**
A1 (active stage 1)	Ulcer with mucus coating and marginal elevation because of oedema
A2 (active stage 2)	Mucus-coated ulcer with discrete margin and less oedema than active stage 1
H1 (healing stage 1)	Healing ulcer < 50% covered by regenerating epithelium with or without covering folds
H2 (healing stage 2)	Ulcer with a mucosal break, but almost covered by regenerating epithelium
S1 (scar stage 1)	Red scar with rough epithelialization and mucosal break(s)
S2 (scar stage 2)	White scar with complete re-epithelialization

English version of the ulcer staging system of Sakita and Fukutomi [11].

The symptoms of all enrolled patients were evaluated at 1 week, 4 weeks, and 8 weeks after ESD.

### Statistical analysis

Data were analyzed using the Mann–Whitney *U*-test. To assess the progress of ulcer healing, Scheffe’s F-test and repeated calculations of analysis of variance were performed as deemed appropriate. All statistical analyses were performed using SPSS Statistics for Windows (version 19; SPSS Inc., Chicago, IL, USA), and instances with *p* of <0.05 were considered to represent statistically significant differences.

## Results

**Figure 1:**
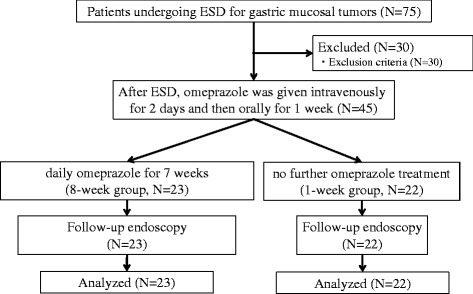
Flow diagram of the study.

A flow diagram shows the disposition of the participants in this study (Figure 1). A total of 75 patients underwent ESD for gastric mucosal tumors between June 2005 and July 2006 at Toyama University Hospital. Of these patients, 45 patients met our inclusion criteria and did not meet any of our exclusion criteria, this including 22 patients who received oral omeprazole therapy for 1 week (1-week group) and 23 patients who received oral omeprazole therapy for 8 weeks (8-week group). The characteristics of the 2 groups are listed in Table 2. No significant difference was noted between the groups with regard to gender, age, smoking habits, *H. pylori* status, carcinoma/adenoma ratio, tumor size, size of resected area, or tumor location.

**Table 2:** **Patient characteristics**

**Variable**	**8-week group (N = 23)**		**1-week group (N = 22)**		P-value*
Male	19	(82.6%)	19	(86.4%)	0.731
Age, years [mean ± standard deviation (SD)]	71.1 ± 7.75		68.2 ± 10.3		0.426
Smoker	5	(21.7%)	5	(22.7%)	0.768
*Helicobacter pylori* positive	16	(69.6%)	18	(81.8%)	0.344
Histopathology					
Adenocarcinoma, W/D or M/D^†^	22	(95.7%)	21	(95.5%)	0.975
Adenoma	1	(4.3%)	1	(4.5%)	
Tumor area, mm^2^ (mean ± SD)	322 ± 317		298 ± 263		0.488
Resected area, mm^2^ (mean ± SD)	1,014 ± 600		1,027 ± 582		1.000
Location					
Upper third	8	(34.8%)	9	(40.9%)	0.807
Mid third	12	(52.2%)	7	(31.8%)	
Lower third	3	(13.0%)	6	(27.3%)	

*Mann–Whitney *U* test, ^†^well-differentiated or moderately differentiated.

The ulcer stages in each group at 4 and 8 weeks after ESD are shown in Table 3. There were no active ulcers in either group at 4 or 8 weeks after ESD. In the 8-week group, the ulcers were stage H2 in 19 patients (83%) and S1 in 4 patients (17%) at 4 weeks; that is, 83% of the patients had unhealed ulcers at this time point. In the 1-week group, the ulcers were stage H1 in 2 patients (9%), H2 in 12 patients (55%), and S1 in 8 patients (36%) at 4 weeks; that is, 64% patients had unhealed ulcers at 4 weeks. At 8 weeks after ESD, the ulcers were stage S (healed) in 22 patients (95.6%) from the 8-week group and in 20 patients (90.9%) from the 1-week group. There was no significant difference in the ulcer-healing rate between the groups at either 4 or 8 weeks after ESD.

**Table 3:** **Ulcer stages in the 2 groups**

			**8-week group N = 23 (%)**		**1-week group N = 22 (%)**		***P*** -value*
Ulcer stage	After 4 weeks	H1	0	(0)	2	(9.1)	0.323
		H2	19	(82.6)	12	(54.5)	
		S1	4	(17.4)	8	(36.4)	
		S2	0	(0)	0	(0)	
	After 8 weeks	H1	0	(0)	0	(0)	1.000
		H2	1	(4.3)	2	(9.1)	
		S1	21	(91.3)	18	(81.8)	
		S2	1	(4.3)	2	(9.1)	

*Mann–Whitney *U* test.

**Figure 2:**
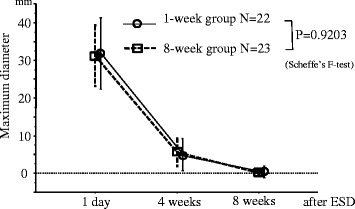
The maximum ulcer diameter (mean ± standard deviation) at 1 day, 4 weeks and 8 weeks after ESD. There were no significant differences between the two groups.

The maximum diameter of the ulcers caused by ESD in both treatment groups at 1 day, 4 weeks, and 8 weeks after ESD were measured endoscopically. The rate of ulcer healing was not significantly different between the groups (Figure 2).

The occurrence of ESD-related abdominal pain and bleeding, which required endoscopic hemostasis after ESD, is shown in Table 4. Between 1 and 7 days after the ESD, 2 patients in the 8-week group and 1 in the 1-week group experienced abdominal pain. No patient reported abdominal pain at 4 or 8 weeks after ESD. Bleeding from the ulcer was observed in 2 patients in the 8-week group and in 1 patient in the 1-week group, as assessed by follow-up endoscopy performed at 1 day after ESD. These patients underwent hemostasis procedures and no bleeding was detected afterwards. No patient had melena, hematemesis, or anemic progression that warranted treatment.

**Table 4:** **ESD-related abdominal pain and bleeding after ESD**

	**8-week group N = 23**		**1-week group N = 22**		P-value*
Patients with pain, N (%)					
Within 1 week	2	(8.6)	1	(4.5)	0.581
	0	(0)	0	(0)	1.000
After 4–8 weeks	0	(0)	0	(0)	1.000
Bleeding, N (%)					
Bleeding^†^	2	(8.6)	1	(4.5)	0.581
Melena, hematemesis or anemia	0	(0)	0	(0)	1.000

*Mann–Whitney *U* test.

^†^Bleeding observed by endoscopy at 1 day after ESD.

## Discussion

This study showed that the quality and rate of healing of artificial ulcers after ESD were not significantly different between groups treated for 1 or 8 weeks with oral omeprazole. This is, to the best of our knowledge, the first report to evaluate an extremely short period (1 week) of oral PPI administration for treating ESD-induced gastric ulcers. PPIs have been generally administered to achieve rapid healing of EMR-induced gastric ulcers [12,13]. In order to accelerate ulcer healing, clot stabilization through elevation of the intragastric pH is required. A bolus intravenous injection of PPI followed by continuous infusion can be effective for decreasing the rate of rebleeding rate in patients who had received successful endoscopic treatment for bleeding peptic ulcers [14]. However, there has been no consensus about whether artificial ulcers and peptic ulcers should be similarly managed. Several studies have suggested that EMR-induced ulcers heal more easily than peptic ulcers. Lee *et al.* compared the healing of small ulcers induced by conventional EMR after 1 and 4 weeks of the PPI treatment and reported that there was no difference in the percentage reduction of ulcer size [15]. Therefore, short-term omeprazole therapy is generally used to treat ulcers after conventional EMR for gastric lesions.

ESD has a major advantage compared with conventional EMR—it allows the *en bloc* resection of small to large tumors. Therefore, ESD has recently become popular in Japan, because of which the size of the artificial ulcers has increased. The mean diameter of a peptic ulcer is usually approximately 10–20 mm, which is similar to the size of the ulcer caused by conventional EMR. Size is an important determinant of the healing of peptic ulcers. Ulcer diameter has been reported to predict healing of a *H. pylori*-related gastric ulcer within 8 weeks [16-18]. One week of triple-therapy against *H. pylori*, including double-dose PPI, was not sufficient treatment for gastric ulcers that were very large (>15 mm), and these ulcers required additional treatment to heal [18]. However, artificial ulcers induced by ESD are usually larger than those induced by conventional EMR, and a larger ulcer increases the risk of bleeding and requires a longer healing time [19]. This suggests that the optimum duration of the PPI treatment after ESD may differ from that after conventional EMR. Therefore, we investigated the efficacy of 2 PPI regimens in this study. In the conventional EMR study of Lee *et al.*, the initial mean [± standard deviation (SD)] ulcer size was 503.8 (±301.6) mm^2^ [15], while the initial mean (±SD) ulcer area was 1027 (±582) mm^2^ in our 1-week group. Although the ulcer area in our study was clearly larger than that in the study of Lee *et al.*, there was no significant difference in the results of the 2 studies. This suggested that the potent acid suppression provided by 1 week of oral omeprazole therapy after 2 days of intravenous omeprazole administration may be sufficient to heal ESD-induced gastric ulcers. This result was quite different from previous studies that showed that the short-term PPI treatment was not sufficient for the healing of larger peptic ulcers [20,21].

Regarding the medication cost, the total cost was 3646 yen for the 1-week group (omeprazole injection for 2 days and tablets for 1 week) and 11,946 yen for the 8-week group (omeprazole injection for 2 days and tablets for 8 weeks). Thus, at only 30% of the cost for the 8-week group, the cost-effectiveness is markedly greater for the 1-week group. It is estimated that approximately 10,000 ESD procedures are performed annually in Japan; therefore, 83,000,000 yen could be saved every year if the duration of omeprazole use could be decreased to 1 week from 8 weeks in patients who undergo ESD.

We have also evaluated *H. pylori* infection status because infection can influence the quality and speed of gastric ulcer healing [22]. Kakushima *et al.* reported that *H. pylori* infection status did not affect the healing of artificial ulcers after ESD [23]. In our study, no correlation was found between *H. pylori* infection status and ulcer healing in either group (data not shown).

Only three patients in this study needed urgent endoscopy for gastric bleeding and the bleeding rate seemed to be lower than previously reported [10]. This may be because of recent improvements of hemostasis techniques performed after ESD. However, this study may have too small of a sample size to robustly evaluate the risk of bleeding after ESD.

## Conclusions

Our results suggested that use of oral omeprazole for 1 week can be sufficient for healing ESD-induced artificial ulcers. The administration of oral omeprazole for 1 week after ESD for gastric lesions should also be considered as it is more cost effective than 8 weeks of treatment. However, a large-scale prospective clinical trial with a statistically determined sample size will be required to confirm these results.

## References

[1] Tada M, Murakami A, Karita M, Yanai H, Okita K (1993). Endoscopic resection of early gastric cancer. Endoscopy.

[2] Soetikno RM, Gotoda T, Nakanishi Y, Soehendra N (2003). Endoscopic mucosal resection. Gastrointest Endosc.

[3] Ono H, Kondo H, Gotoda T, Shirao K, Yamaguchi H, Saito D (2001). Endoscopic mucosal resection for treatment of early gastric cancer. Gut.

[4] Lightdale CJ (2004). Endoscopic mucosal resection: this is our turf. Endoscopy.

[5] Rosch T, Sarbia M, Schumacher B, Deinert K, Frimberger E, Toermer T (2004). Attempted endoscopic en bloc resection of mucosal and submucosal tumors using insulated-tip knives: a pilot series. Endoscopy.

[6] Miyamoto S, Muto M, Hamamoto Y, Boku N, Ohtsu A, Baba S (2002). A new technique for endoscopic mucosal resection with an insulated-tip electrosurgical knife improves the completeness of resection of intramucosal gastric neoplasms. Gastrointest Endosc.

[7] Hashimoto T, Adachii K (1997). Changes in gastric mucosal blood flow during healing of EMR-induced ulcer: comparison with peptic ulcer. Dig Endosc.

[8] Kakushima N, Yahagi N, Fujishiro M, Iguchi M, Oka M, Kobayashi K (2004). The healing process of gastric artificial ulcers after endoscopic submucosal dissection. Dig Endosc.

[9] Lee SH, Lee CK, Chung IK, Shim YS, Lee TH, Lee SH (2012). Optimal duration of proton pump inhibitor in the treatment of endoscopic submucosal dissection-induced ulcers: a retrospective analysis and prospective validation study. Dig Dis Sci.

[10] Muraki Y, Enomoto S, Iguchi M, Fujishiro M, Yahagi N, Ichinose M (2012). Management of bleeding and artificial gastric ulcers associated with endoscopic submucosal dissection. World J Gastrointest Endosc.

[11] Sakita T, Fukutomi H, Yoshitoshi Y (1971). Endoscopic diagnosis. Ulcer of stomach and duodenum [Japanese].

[12] Yamaguchi Y, Katsumi N, Tauchi M, Toki M, Nakamura K, Aoki K (2005). A prospective randomized trial of either famotidine or omeprazole for the prevention of bleeding after endoscopic mucosal resection and the healing of endoscopic mucosal resection-induced ulceration. Aliment Pharmacol Ther.

[13] Ye BD, Cheon JH, Choi KD, Kim SG, Kim JS, Jung HC (2006). Omeprazole may be superior to famotidine in the management of iatrogenic ulcer after endoscopic mucosal resection: a prospective randomized controlled trial. Aliment Pharmacol Ther.

[14] Lau JY, Sung JJ, Lee KK, Yung MY, Wong SK, Wu JC (2000). Effect of intravenous omeprazole on recurrent bleeding after endoscopic treatment of bleeding peptic ulcers. N Engl J Med.

[15] Lee SY, Kim JJ, Lee JH, Kim YH, Rhee PL, Paik SW (2004). Healing rate of EMR-induced ulcer in relation to the duration of treatment with omeprazole. Gastrointest Endosc.

[16] Arkkila PE, Seppälä K, Kosunen TU, Haapiainen R, Kivilaakso E, Sipponen P (2003). Eradication of Helicobacter pylori improves the healing rate and reduces the relapse rate of nonbleeding ulcers in patients with bleeding peptic ulcer. Am J Gastroenterol.

[17] Treiber G, Lambert JR (1998). The impact of Helicobacter pylori eradication on peptic ulcer healing. Am J Gastroenterol.

[18] Higuchi K, Fujiwara Y, Tominaga K, Watanabe T, Shiba M, Nakamura S (2003). Is eradication sufficient to heal gastric ulcers in patients infected with Helicobacter pylori? A randomized, controlled, prospective study. Aliment Pharmacol Ther.

[19] Oka S, Tanaka S, Kaneko I, Mouri R, Hirata M, Kawamura T (2006). Advantage of endoscopic submucosal dissection compared with EMR for early gastric cancer. Gastrointest Endosc.

[20] Terano A, Arakawa T, Sugiyama T, Suzuki H, Joh T, Yoshikawa T (2007). Rebamipide, a gastro-protective and anti-inflammatory drug, promotes gastric ulcer healing following eradication therapy for Helicobacter pylori in a Japanese population: a randomized, double-blind, placebo-controlled trial. J Gastroenterol.

[21] Hiraishi H, Haruma K, Miwa H, Goto H (2010). Clinical trial: irsogladine maleate, a mucosal protective drug, accelerates gastric ulcer healing after treatment for eradication of Helicobacter pylori infection—the results of a multicentre, double-blind, randomized clinical trial (IMPACT study). Aliment Pharmacol Ther.

[22] Graham DY, Lew GM, Klein PD, Evans DG, Evans DJ, Saeed ZA (1992). Effect of treatment of Helicobacter pylori infection on the long-term recurrence of gastric or duodenal ulcer. A randomized, controlled study. Ann Intern Med.

[23] Kakushima N, Fujishiro M, Yahagi N, Kodashima S, Nakamura M, Omata M (2006). Helicobacter pylori status and the extent of gastric atrophy do not affect ulcer healing after endoscopic submucosal dissection. J Gastroenterol Hepatol.

